# Asymptomatic mature intrapericardial teratoma in an adult: a case report of a rare condition

**DOI:** 10.1186/s40792-024-01902-0

**Published:** 2024-04-28

**Authors:** Hikaru Watanabe, Naoki Kanauchi, Jun Suzuki, Soumei Matsuo, Satoshi Shiono

**Affiliations:** 1 Department of Surgery II, Faculty of Medicine, Yamagata University, 2-2-2 Iida-Nishi, Yamagata, Yamagata Japan; 2 Department of General Thoracic Surgery, Nihonkai General Hospital, Sakata, Yamagata Japan

**Keywords:** Adult, Intrapericardial tumor, Mature teratoma, Surgery

## Abstract

**Background:**

Benign mature teratomas are the most common type of anterior mediastinal germ cell tumor. Mature intrapericardial teratomas are generally diagnosed during infancy because of symptoms of cardiac compression. In contrast, mature adult intrapericardial teratomas are extremely rare, accounting for less than 1% of mature intrapericardial teratomas. We describe herein a case of a mature intrapericardial teratoma in an asymptomatic adult.

**Case presentation:**

A 52-year-old woman was found by computed tomography during a health checkup to have an anterior mediastinal mass. She was asymptomatic and hemodynamically stable with no evidence of heart failure. The preoperative provisional radiological diagnosis was a mature intrapericardial teratoma. A median sternotomy revealed an approximately 5-cm diameter protruding intrapericardial mass with a smooth surface. The mass was completely resected. Histopathological examination resulted in a diagnosis of a mature intrapericardial teratoma. The patient did well and has no evidence of recurrence 5 years after surgery.

**Conclusions:**

Mature intrapericardial teratomas in adults are extremely rare. Given the risks of malignant transformation, rupture, compression of the heart, and infection, excision is indicated to prevent development of serious manifestations.

## Background

Teratomas, which are tumors of embryonic origin, are composed of various proportions of elements derived from the three germinal layers [1]. Tumors in which more than 50% of elements are well-differentiated are referred to as mature teratomas. Intrapericardial teratoma are an uncommon type of teratoma. Intrapericardial teratomas are generally diagnosed during infancy because they typically manifest various symptoms and signs. In contrast, intrapericardial teratomas in adults are unusual, the reported incidence being < 1% of all mature intrapericardial teratomas [2, 3]. Herein, we report an adult with a mature, asymptomatic, intrapericardial teratoma.

## Case report

**Fig. 1:**
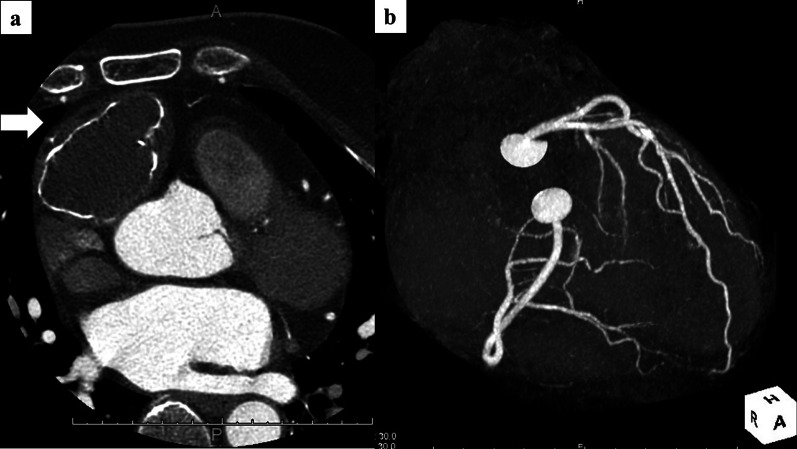
**a** Chest computed tomography image showing a 50 × 45 × 40 mm mass in the anterior mediastinum with irregular marginal calcification. **b** Coronary CT shows no coronary aneurysm or feeding artery from the coronary artery

**Fig. 2:**
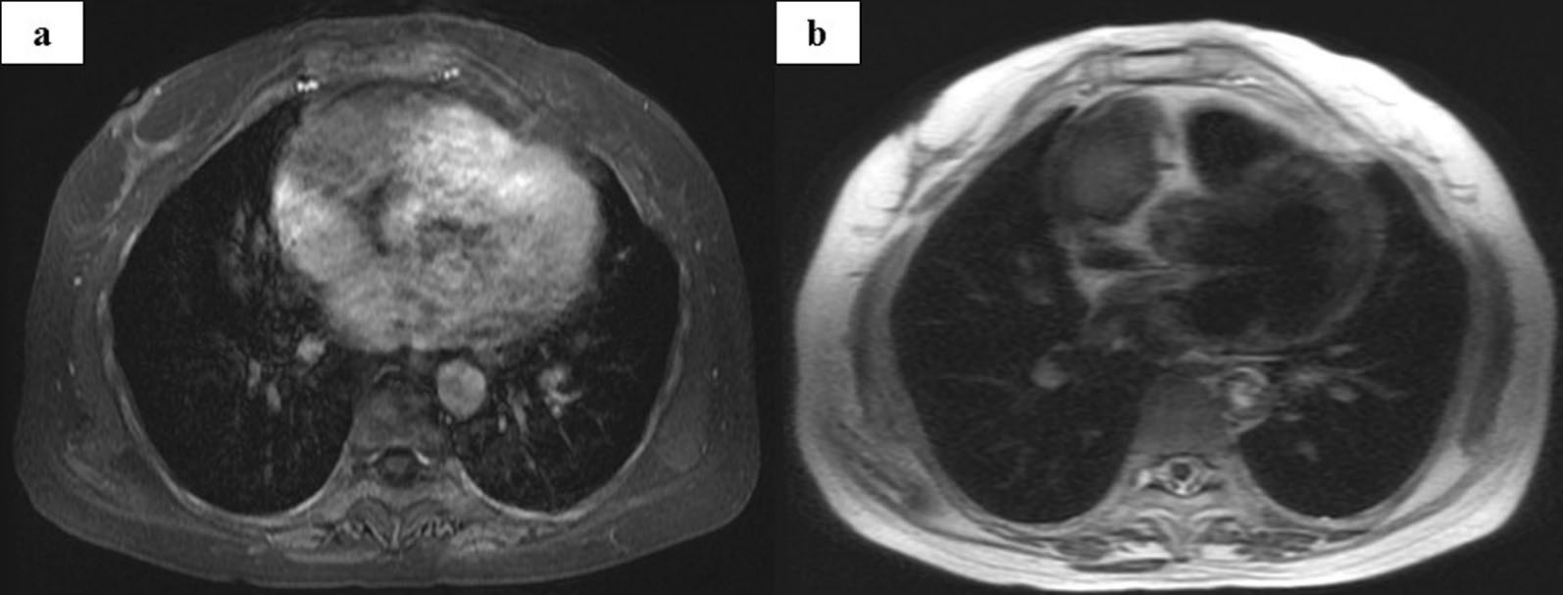
Chest magnetic resonance images showing a mass in the anterior mediastinum on **a** T1-weighted and **b** T2-weighted sequences

A 52-year-old woman presented because an anterior mediastinal mass had been discovered by computed tomography (CT) during a health checkup. She was healthy and had never experienced chest pain, dyspnea, or palpitations. Physical examination revealed no evidence of neuromuscular disorders or heart failure. Routine blood biochemistry was normal. Chest CT revealed an anterior mediastinal mass with central intermediate attenuation and irregular marginal calcification (Fig. 1a). Coronary CT did not demonstrate a coronary aneurysm or a feeding artery from the coronary artery (Fig. 1b). Cardiac magnetic resonance imaging (MRI) showed that this tumor was located within the pericardial sac adjacent to the right atrium with hypodensities on both T1- and T2-weighted sequences indicating soft inclusions. There was no finding of the tumor to invade or adhere to the heart in preoperative chest MRI. (Fig. 2a, b). We did not perform a transthoracic needle biopsy because of the risks of dissemination or hemorrhagic complications.

**Fig. 3:**
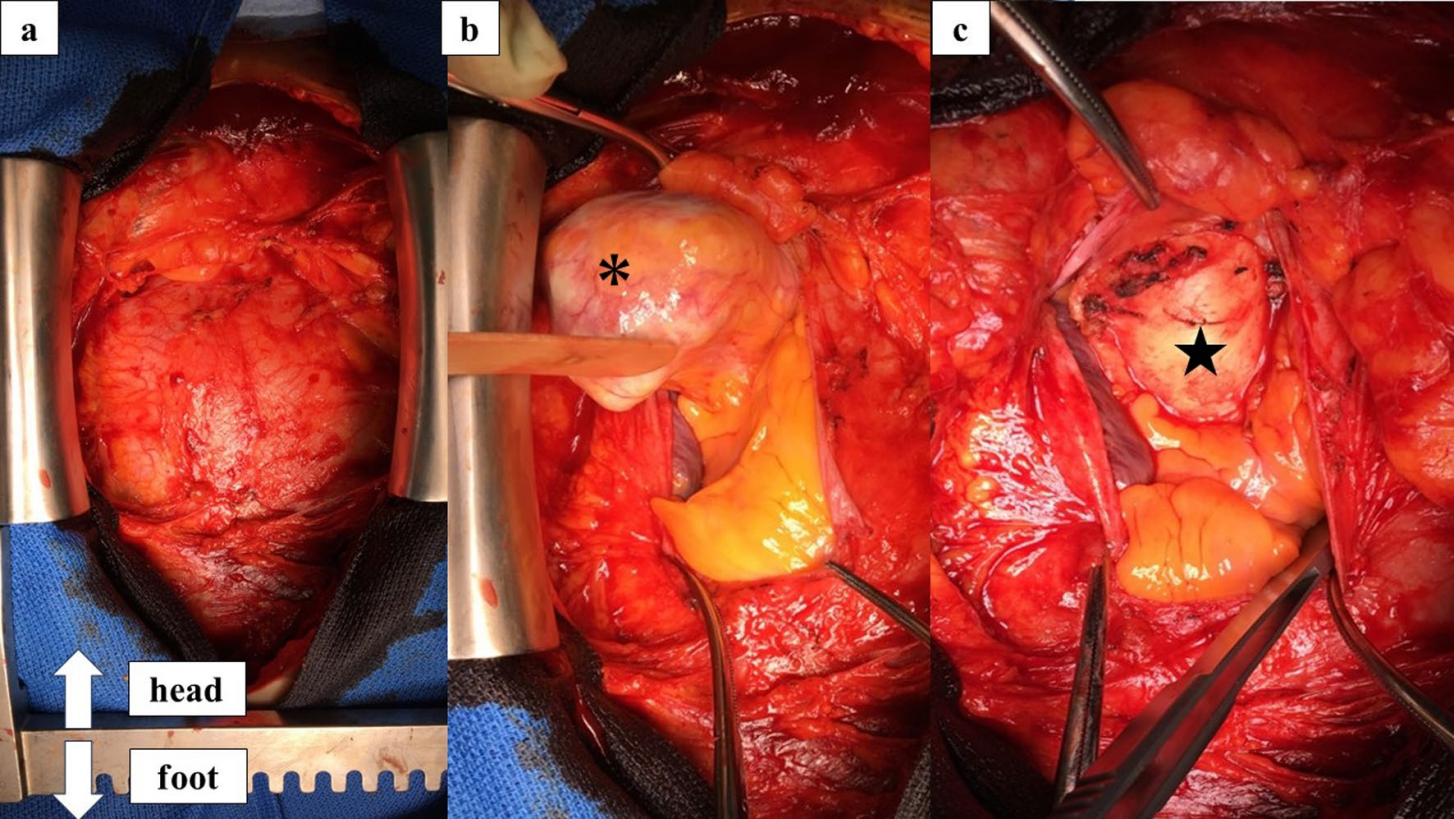
**a** Before the pericardiotomy. **b** Intraoperative photograph showing the tumor is attached to the adventitia of ascending aorta. **c** Surgical field after tumor resection. (*) Tumor and (★) aortic adventitia at the root of the ascending aorta

**Fig. 4:**
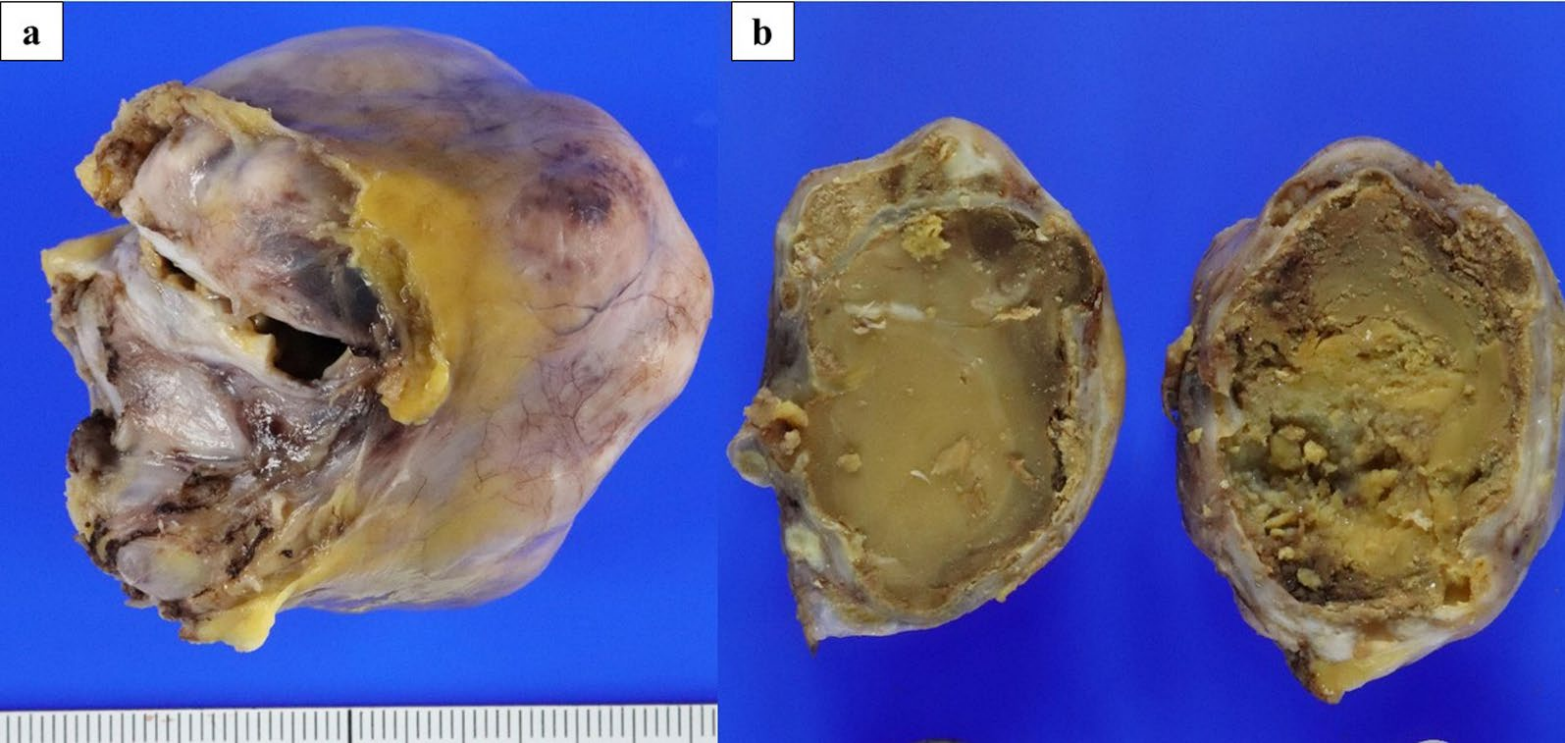
**a** Gross specimen: a bosselated intrapericardial mass measuring 5.2 × 5.0 × 5.0 cm with a smooth external surface. **b** Macroscopic examination revealed a large cyst containing yellow cheese-like and tan-brown muddy material surrounded by calcification

With a provisional preoperative diagnosis of intrapericardial teratoma, surgery was performed via a median sternotomy without cardiopulmonary bypass because there was no evidence of tumor invasion of the heart (Fig. 3a). Pericardiotomy exposed the tumor within the pericardial sac and revealed that the lesion was attached to the aortic adventitia at the root of the ascending aorta (Fig. 3b, c). The tumor had no feeding artery and was completely resected. Intraoperatively, the tumor was found to consist of an encapsulated, cystic, intrapericardial, soft-tissue mass measuring 5.2 × 5.0 × 5.0 cm and weighing 45 g (Fig. 4a). Macroscopically, the lesion mainly comprised a large cyst containing yellow and white cheese-like and tan-brown muddy material surrounded by calcification (Fig. 4b). Pathological finding showed that this tumor was made of mature cartilage, bone, bronchial epithelium and nerves. A histological diagnosis of a mature cystic teratoma was made without immature components and there was no morphological evidence of malignancy in the tumor. The postoperative course was uneventful; the patient recovered fully and was discharged on postoperative day 5. There is no evidence of recurrence 5 years after surgery.

This case report was approved by the Ethics Committee of the Faculty of Medicine, Nihonkai General Hospital (005-6-15) and the patient gave written, informed consent to publication of this report.

## Discussion

Mature intrathoracic teratomas are typically located in the anterior mediastinum. They account for 10% of congenital teratomas and 7% of all pediatric germ cell tumors. Thus, intrathoracic teratomas, including intrapericardial or pericardial teratomas, are extremely rare [4]. Histologically, anterior mediastinal teratomas have associated thymic tissue around the tumor, leading to Schlumberger’s theory of intra-thymic tissue invasion as a possible pathogenesis of mediastinal teratomas [5]. However, there are no report of thymic tissue in intrapericardial teratomas. Friedman's theory of germ cell intrusion is also considered a strong theory considering that teratomas occur in a variety of sites other than the mediastinum, including the pineal gland, retroperitoneum, sacral region, and gonads [6]. In the respect of embryological point, the difference between intrapericardial and extrapericardial teratoma is not certain. Mature intrapericardial teratomas are typically diagnosed during infancy because they manifest in a variety of ways, including pericardial effusion, dyspnea, arrhythmias, respiratory distress, and hypotension [7, 8]. Intrapericardial teratomas rarely present in adults [9, 10], with, to the best of our knowledge, only five cases of intrapericardial teratoma in adults having been reported [3, 9–12] (Table 1). Four of them were symptomatic, having chest pain, cough, dyspnea, and palpitations secondary to pericardial tamponade and compression of right-sided heart structures. Anterior mediastinal teratomas can rupture, causing cardiac tamponade [10, 11]. Mature teratomas of the mediastinum, which are usually benign, often grow slowly. Thus, our patient had never experienced symptoms of an enlarging intrapericardial mass and her teratoma was discovered incidentally.

**Table 1:** Reported surgical cases of intrapericardial mature teratoma in an adult

Case	Author	Year	Age	Gender	Symptom	Tumor size (cm)	Location	Feeding artery	Cystic change
1	Bitar [9]	1998	26	Male	Chest pain and fever	19.0	Aortic adventitia at the root of the ascending aorta	−	+
2	Brown [10]	2006	44	Male	Chest pain and dyspnea	Unknown	Unknown due to pericardial effusion	Unknown	+
3	Singh [11]	2009	16	Female	Dry cough, fever and shortness of breath	7.0	Pericardium near the left main pulmonary artery	−	−
4	Gonzalez [3]	2010	51	Female	Asymptomatic	9.5	Aortic adventitia at the root of the ascending aorta	From the aorta	+
5	Cohen [12]	2013	50	Male	Palpitation	7.0	Attached to the greater curvature of the ascending aorta	−	+
6	Our case	2024	51	Female	Asymptomatic	5.2	Aortic adventitia at the root of the ascending aorta	−	+

A CT scan can identify the location and characteristics of a thoracic tumor, including a mature intrapericardial teratoma. Additionally, MRI is a mandatory modality for assessing pericardial involvement and the relationship between the tumor and surrounding vascular structures [10, 13]. The diagnosis is typically made by pathological evaluation of the resected specimen. The role of transthoracic needle biopsy in debatable. A needle biopsy can cause serious complications and often fails to yield sufficient tissue for an accurate diagnosis [3]. To our knowledge, there are no published reports concerning the usefulness of transthoracic needle biopsy for intrapericardial teratomas. When intrapericardial teratoma is the provisional diagnosis, transthoracic needle biopsy should not be performed because of the risk of complications.

Complete resection of mature teratomas is recommended, including intrapericardial ones. Incomplete resection of benign teratomas to relieve compressive symptoms is indicated if the tumor cannot be excised completely because this would endanger surrounding vital structures. The prognosis of intrapericardial teratomas after complete resection is favorable [14].

There are several reasons for excising intrapericardial teratomas. First, they carry a risk of malignant transformation [15]. Second, intrapericardial teratomas have the potential to rupture and perforate. There are two previous reports of intrapericardial teratoma perforating into the pericardial sac (8, 9). When the tumor perforates into the pericardial sac and causes inflammation, tumor resection becomes more difficult owing to severe adhesion. Third, when intrapericardial, teratomas can result in constrictive pericarditis [16]. Finally, blood supply of these tumor is reportedly derived from the adventitial vessels of the aorta, creating a risk of massive hemorrhage from the aorta during dissection [3]. When dissecting the aortic adventitia, it is necessary to ascertain whether or not feeding arteries are present. To evaluate the blood supply of these tumor, preoperative coronary CT could be informative.

## Conclusions

Intrapericardial teratomas are rare benign tumors, may be life-threatening, and should be resected when diagnosed, even in asymptomatic patients.

## Data Availability

Not applicable.

## Abbreviations

CT: Computed tomography
MRI: Magnetic resonance imaging
